# Isolation and pathogenicity comparison of two novel natural recombinant porcine reproductive and respiratory syndrome viruses with different recombination patterns in Southwest China

**DOI:** 10.1128/spectrum.04071-23

**Published:** 2024-03-21

**Authors:** Bingzhou Huang, Lishuang Deng, Tong Xu, Zhijie Jian, Siyuan Lai, Yanru Ai, Zhiwen Xu, Ling Zhu

**Affiliations:** 1 College of Veterinary Medicine, Sichuan Agricultural University, Chengdu, China; 2 Key Laboratory of Animal Diseases and Human Health of Sichuan Province, Chengdu, China; Oklahoma State University College of Veterinary Medicine, Stillwater, Oklahoma, USA

**Keywords:** PRRSV, isolation, recombination, lineage 1.8, lineage 8, pathogenicity

## Abstract

Porcine reproductive and respiratory syndrome virus (PRRSV) causes significant economic losses in the swine industry. Frequent mutations and recombinations account for PRRSV immune evasion and the emergence of novel strains. In this study, we isolated and characterized two novel PRRSV-2 strains from Southwest China exhibiting distinct recombination patterns. They were designated SCABTC-202305 and SCABTC-202309. Phylogenetic results indicated that SCABTC-202305 was classified as lineage 8, and SCABTC-202309 was classified as lineage 1.8. Amino acid mutation analysis identified unique amino acid substitutions and deletions in ORF5 and Nsp2 genes. The results of the recombination analysis revealed that SCABTC-202305 is a recombinant with JXA1 as the major parental strain and NADC30 as the minor parental strain. At the same time, SCABTC-202309 is identified as a recombinant with NADC30 as the major parental strain and JXA1 as the minor parental strain. In this study, we infected piglets with SCABTC-202305, SCABTC-202309, or mock inoculum (control) to study the pathogenicity of these isolates. Although both isolated strains were pathogenic, SCABTC-202305-infected piglets exhibited more severe clinical signs and higher mortality, viral load, and antibody response than SCABTC-202309-infected piglets. SCABTC-202305 also caused more extensive lung lesions based on histopathology. Our findings suggest that the divergent pathogenicity observed between the two novel PRRSV isolates may be attributed to variations in the genetic information encoded by specific genomic regions. Elucidating the genetic determinants governing PRRSV virulence and transmissibility will inform efforts to control this devastating swine pathogen.

**IMPORTANCE:**

Porcine reproductive and respiratory syndrome virus (PRRSV) is one of the most critical pathogens impacting the global swine industry. Frequent mutations and recombinations have made the control of PRRSV increasingly difficult. Following the NADC30-like PRRSV pandemic, recombination events involving PRRSV strains have further increased. We isolated two novel field PRRSV recombinant strains, SCABTC-202305 and SCABTC-202309, exhibiting different recombination patterns and compared their pathogenicity in animal experiments. The isolates caused higher viral loads, persistent fever, marked weight loss, moderate respiratory clinical signs, and severe histopathologic lung lesions in piglets. Elucidating correlations between recombinant regions and pathogenicity in these isolates can inform epidemiologic tracking of emerging strains and investigations into viral adaptive mechanisms underlying PRRSV immunity evasion. Our findings underscore the importance of continued genomic surveillance to curb this economically damaging pathogen.

## INTRODUCTION

Porcine reproductive and respiratory syndrome (PRRS) was first detected in North America in 1987 and has since emerged as an economically significant disease affecting the global swine industry (1). The etiological agent, PRRS virus (PRRSV) is a 15.4-kb enveloped, positive-sense, single-stranded RNA virus that belongs to the genus *Porartevirus*, family *Arteriviridae*, and order *Nidovirales* (2). The PRRSV genome comprises 10 open reading frames (ORFs): ORF1a, ORF1b, ORF2a, ORF2b, ORF3, ORF4, ORF5, ORF5a, ORF6, and ORF7. Among these, ORF1a and ORF1b encode the replicase polyproteins pp1a and pp1b, respectively, which undergo autoproteolytic processing to generate 14 nonstructural proteins (Nsps): Nsp1α, Nsp1β, Nsp2 (Nsp2Tf and Nsp2N), Nsp3-6, Nsp7α, Nsp7β, and Nsp8-12 (3, 4).

PRRSV can be classified into two genotypes: PRRSV-1, represented by the prototype strain Lelystad, and PRRSV-2, represented by the prototype strain VR-2332. These two genotypes differ by approximately 60% at the nucleotide level (5). Shi et al. (1) constructed a global classification system for PRRSV based on a comprehensive analysis of complete ORF5 gene sequences. This system further delineates PRRSV-2 into four lineages: lineage 1 (NADC30-like), lineage 3 (QYYZ-like), lineage 5 (VR2332-like), and lineage 8 (JXA1-like and CH-1a-like) (1). PRRSV-2 has predominated in China since emerging in the late 1990s, undergoing continuous diversification at a relatively rapid evolutionary pace (6). In 2006, an outbreak of highly pathogenic PRRSV (HP-PRRSV) belonging to sublineage 8.7 resulted in over 2 million pig fatalities (7). Although the modified live virus vaccine (MLV) temporarily curbed HP-PRRSV outbreaks, the genetic heterogeneity and rapid mutation rate of PRRSV-2 enables viral immune evasion, impeding sustained control (6). During 2013–2014, circulation of NADC30-like PRRSV strains (lineage 1.8) was documented across several provinces in China, with these viruses gradually becoming epidemiologically predominant owing to their increased recombination and mutation frequencies (7). First identified in China in 2017, NADC34-like PRRSV (lineage 1.5) has since disseminated rapidly nationwide (8). The co-circulation of multiple PRRSV genotypes in Chinese swine herds has caused substantial economic damage to the pork industry while intensifying prospects for viral recombination.

The rapid evolution and recombination of PRRSV have caused the emergence of novel, highly virulent viruses that induce clinical PRRS outbreaks (9). In recent years, researchers have reported the emergence of various novel recombinant PRRSV strains derived from NADC30-like PRRSV outbreaks exhibiting different pathogenicity in China (7, 10, 11). Previous studies have indicated that NADC30-like and JXA1-like PRRSV recombinant strains manifest great virulence, approximating that of JXA1-like strains, frequently eliciting abortion, stillbirth, and neonatal mortality in sow herds (12–14).

In this study, we isolated two novel recombinant PRRSV strains (SCABTC-202305 and SCABTC-202309) from suspected PRRSV-infected swine farms in Southwest China. To further identify these isolates, we determined their complete genome sequences and evaluated pathogenicity in piglets. Phylogenetic analysis classified SCABTC-202305 as lineage 8, and SCABTC-202309 as lineage 1.8. Recombination analysis revealed that two isolates in this study exhibited different recombination patterns. Furthermore, we evaluated their virulence properties in 1-week-old piglets, and SCABTC-202305-infected piglets exhibited relatively more severe clinical signs and higher mortality, viral load, antibody response, and lung lesions compared to SCABTC-202309-infected piglets. Our study suggests that differences in specific genomic regions may contribute to the pathogenicity and epidemic capacity.

## MATERIALS AND METHODS

### Isolation and identification of PRRSV

Clinical serum samples were collected from suspected PRRSV-infected, unvaccinated piglets in two independent swine farms in Southwest China. The affected pigs exhibited high fever, respiratory distress, and mortality rates of 18.52% (75/405) and 8.49% (79/930) in Sichuan and Guizhou, respectively (Table 1). Following filtration of PRRSV-positive sera through 0.22-µm membranes, 1-mL aliquots were aseptically inoculated onto Marc-145 cells and were incubated for 1 hour (37°C, 5% CO_2_). The inoculated cells were subsequently propagated in Dulbecco modified Eagle medium (DMEM; Thermo Fisher, Shanghai, China) supplemented with 3% Gibco fetal bovine serum (FBS; Thermo Fisher, Shanghai, China) for 3 days and were monitored for cytopathic effects (CPE). Upon CPE manifestation, the infected cells underwent two freeze-thaw cycles at −80°C, centrifugation at 5,000 *g* for 5 minutes, and collection of viral supernatants. The viral supernatants were utilized to infect Marc-145 cells and were serially passaged five times. PRRSV was detected by real-time quantitative PCR (RT-qPCR) in the supernatant from each viral passage. PRRSV RNA in testis was quantified by established qRT-PCR (15). cDNA was reverse transcribed from 1-µg total RNA using the PrimeScript RT Reagent Kit (Takara, Beijing, China). Primers were 5′-GCAAGTACATTCTGGCCCCT-3′ and 5′-CAATGTGCCGTTGACCGTAG-3′. Cycling conditions were 95°C for 30 seconds, then 40 cycles of 95°C for 5 seconds and 60°C for 20 seconds. PRRSV RNA was normalized to a housekeeper gene for relative quantification.

**TABLE 1:** Information on the source farm of the isolated strain

Strains	SCABTC-202305	SCABTC-202309
Collection year	March 2023	June 2023
Isolation region	Yaan, Sichuan, China	Zunyi, Guizhou, China
Pig farm size	About 600 sows and 5,000 piglets	About 70 sows and 500 piglets
Infectious history	Never been infected with PRRSV	Previously infected with PRRSV (NADC30-like) in 2023.02
Vaccination history	Never had the PRRSV vaccine	Previously vaccinated against PRRSV (MLV) in 2023.03
Clinical signs	Sow abortion rate of 20.06%, piglet mortality of 18.52%	Sow abortion rate of 9.72%, piglet mortality of 8.49%
Samples	Serum	Serum
GenBank accession	OR365672	OR766560

Indirect immunofluorescence assay (IFA) was conducted using a PRRSV N protein-specific monoclonal antibody (GeneTex, California, USA) and fluorescein isothiocyanate (FITC)-conjugated secondary antibody (Proteintech, Wuhan, China) to detect PRRSV. In detail, Marc-145 cells were infected with PRRSV and were incubated for 48 hours. The cells were fixed in 4% paraformaldehyde for 10 minutes and were permeabilized with 0.2% Triton X-100 for 5 minutes. After blocking with 5% bovine serum albumin (BSA) at 37°C for 1 hour, the cells were incubated with PRRSV N protein-specific monoclonal antibody (1:500 dilution) overnight at 4°C. Then, the cells were incubated with FITC-conjugated secondary antibody (1:1,000 dilution) at 37°C for 1 hour. Nuclei were counterstained with 4′,6-diamidino-2-phenylindole. Cells presenting green fluorescence in the cytoplasm under fluorescence microscopy were counted as positive.

The isolates underwent three rounds of plaque purification followed by six passages in MARC-145 cells and complete genomic sequencing. Virus titers were determined by the Reed-Muench method. Viral samples were sent to Chengdu Lilai Biotechnology Co., Ltd, for transmission electron microscopy photography.

### Whole genome sequencing and genetic analysis

Total RNA was extracted from PRRSV-infected cell suspensions using RNAiso Plus (Takara, Kyoto, Japan) according to the manufacturer’s instructions. Subsequently, the RNA samples were submitted to Shanghai Tanpu Biotechnology Co., Ltd, for whole genome sequencing (WGS).

To investigate the evolutionary relationship of SCABTC-202305 and SCABTC-202309, sequence alignments and phylogenetic analyses were conducted using 41 representative PRRSV strains from GenBank (Table S1). Nucleotide sequences were aligned employing the ClustalX tool in MEGA version 7.0 (NIH, Maryland, USA) (16). Maximum likelihood phylogenetic trees were constructed using 1,000 bootstrap replicates and were compared to the complete gene sequences of PRRSV reference strains in GenBank (17).

### Amino acid mutation analysis

To characterize amino acid mutations in SCABTC-202305 and SCABTC-202309, we employed the ClustalW module in DNAStar software version 7.0 (DNASTAR Inc., Wisconsin, USA) (18) to compare the amino acid sequences of the PRRSV ORF5 and Nsp2 genes to determine sequence similarity.

### Recombination analysis

Potential recombination events were identified using RDP (19), GENECONV (20), BootScan (21), MaxChi (22), Chimera (23), SiScan (24), and 3Seq (25) methods in RDP version 4.1.101 (UCT, Cape Town, South Africa). A recombination event was determined to be authentic if ≥5 algorithms detected a recombination event with *P* < 0.05. For each region, evolutionary analysis of maximum likelihood was performed in MEGA version 7.0 (NIH, Maryland, USA) (26). To visualize the recombinant signal and inferred breakpoint locations, a similarity analysis between the presumptive recombinant sequences and the parental lineages was implemented in SimPlot version 3.5.1 (ReduSoft Ltd., Bad Waldsee, Germany) (27).

### Animal challenge experiment

To evaluate the pathogenicity of SCABTC-202305 and SCABTC-202309, 15 1-week-old PRRSV, pseudorabies virus (PRV), porcine circovirus type 2 (PCV2), classical swine fever virus (CSFV)-seronegative piglets were purchased from a farm in Chengdu. The piglets were randomly divided into three groups and were housed in separate rooms with appropriate care and feeding throughout the experiment.

The challenge experiment commenced following an acclimatization period. Group 1 (*n* = 5) received 2 mL (2 × 10^5^ TCID_50_/mL) of SCABTC-202305 intranasally. Group 2 (*n* = 5) received 2 mL (2 × 10^5^ TCID_50_/mL) of SCABTC-202307 intranasally. The control group (*n* = 5) was sham-inoculated with DMEM medium intranasally. Rectal temperatures, body weights, survival rates, and clinical manifestations were documented daily for each group. Clinical scores were evaluated utilizing a 0–20 scale (28) based on the percentage of lung area affected. Serum, nasal, throat, and anal swabs were collected at 0, 3, 5, 7, and 14 days post-inoculation (dpi). Viral loads in serum, nasal, oropharyngeal, and rectal swabs were detected using RT-qPCR to assess viremia and patterns of viral excretion. Specific antibodies against the N protein of PRRSV in serum were detected using the IDEXX HerdChek PRRS X3 enzyme-linked immunosorbent assay (ELISA) kit (IDEXX, Shanghai, China). After 14 dpi, all surviving piglets were euthanized by an overdose of sodium pentobarbital (Sinopharm, Beijing, China) dosed by intravenous route, and a complete necropsy was performed. The animal studies were approved by the Sichuan Agricultural University Laboratory Animal Management Committee (Approval Number SYXK2019-187). The studies were conducted in accordance with the local legislation and institutional requirements. Written informed consent was obtained from the owners for the participation of their animals in this study.

### Serological and viremia test

Viral loads in serum, nasal, oropharyngeal, and rectal swabs were quantified by RT-qPCR to evaluate viremia and viral shedding patterns. Serum antibodies specific to PRRSV nucleocapsid protein were detected using the IDEXX HerdChek PRRS X3 ELISA kit (IDEXX, Shanghai, China). Upon necropsy, heart, liver, spleen, lung, kidney, inguinal lymph node, and testicular tissue samples were collected from piglets. The viral load in each organ was then measured by qRT-PCR.

### Gross lung pathological and histopathology examination

Gross lung pathology was performed postmortem on pigs that died during the trial, or necropsy was conducted on surviving pigs at 14 dpi. The scoring system applied has been previously described (29). Each piglet received a score from 0 to 100, with each lung lobe assigned a number reflecting its approximate percentage of total lung volume. The right cranial lobe, right middle lobe, cephalic portion of the left cranial lobe, and caudal portion of the left cranial lobe each received 10 points, while the accessory lobes received 5 points. The right and left caudal lobes each received 27.5 points, totaling 100 points for the entire lung.

Lung specimens were fixed in 4% paraformaldehyde, embedded in paraffin, sectioned, and stained with hematoxylin and eosin (H&E). A blinded observer then examined the histopathological changes in the stained lung sections under light microscopy. Lesion severity was scored as 0 (no microscopic lesions), 1 (mild, focal to multifocal interstitial pneumonia), 2 (moderate, multifocal to coalescing interstitial pneumonia), 3 (severe, patchy to coalescing and extensive interstitial pneumonia), or 4 (severe and diffuse interstitial pneumonia) (29).

### Immunohistochemical (IHC) analyses

IHC staining was performed on the lung sections. Tissue sections were treated with 3% H_2_O_2_ solution (pH 7.6) for 10 minutes and then with 5% BSA (Thermo Fisher, Shanghai, China) for 30 minutes. The sections were then incubated overnight at 4°C with a rabbit monoclonal antibody against PRRSV nucleocapsid protein (1:500; Chixun, Guangzhou, China) as the primary antibody. The sections were washed three times with phosphate-buffered saline (PBS) and were incubated at 37°C for 30 min with biotin-conjugated affinity-purified goat anti-rabbit IgG (Proteintech, Wuhan, China) as the secondary antibody. After further incubation with streptavidin-biotin complexes (Boster, Wuhan, China) at 37°C for 30 minutes, the specific binding to the sections was visualized using diaminobenzidine (Boster, Wuhan, China). The percentage of positive cells was calculated for each section using the ImageJ software version 1.5.1 (NIH, Maryland, USA).

### Statistical analysis

Experimental data were analyzed for significance using GraphPad Prism version 8.0.2 (Graphpad Software, California, USA) and are expressed as mean ± standard deviation. *P* < 0.05 was considered significant, and *P* < 0.01 or *P* < 0.001 was considered highly significant.

## RESULTS

### Virus isolation and identification

**Fig 1:**
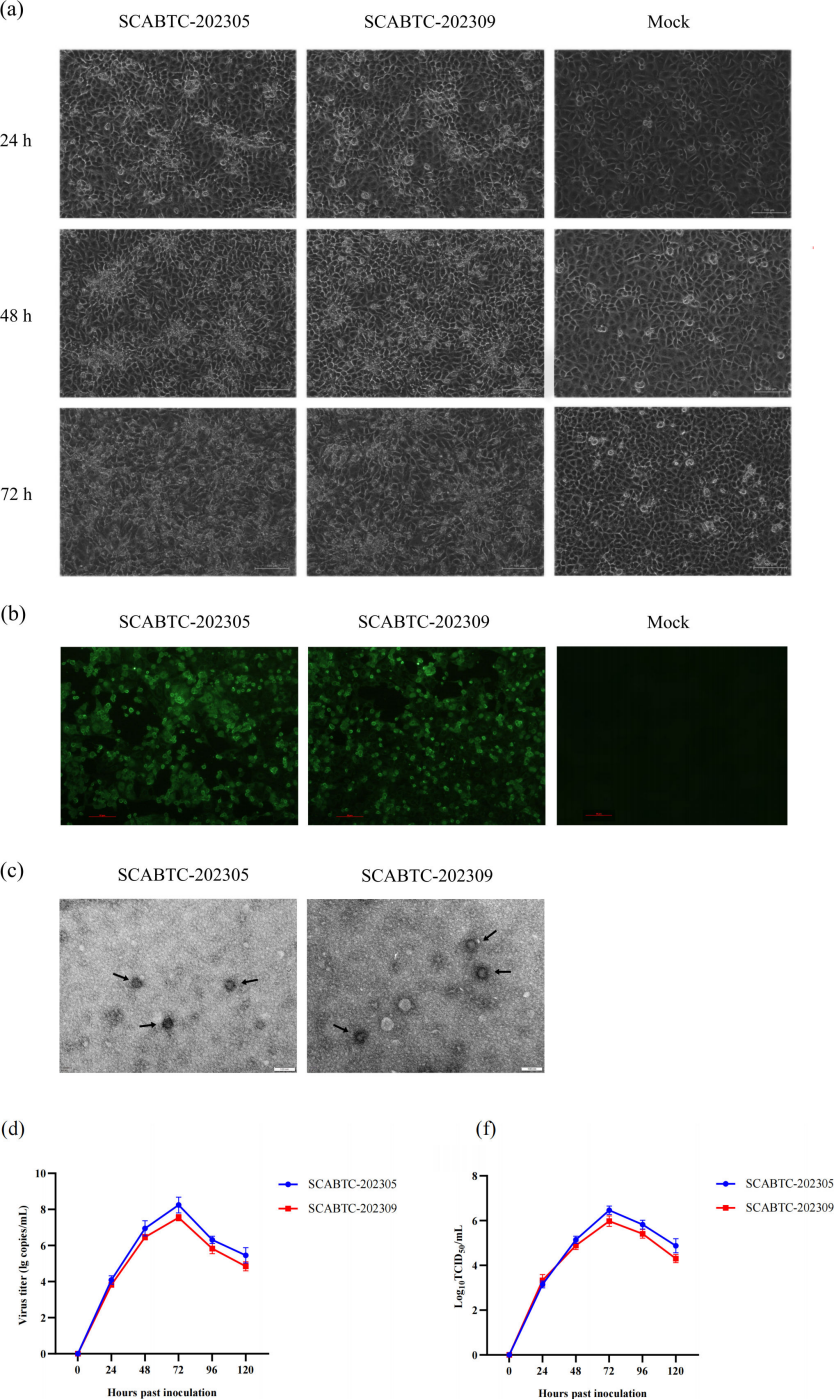
(**a**) Marc-145 CPE at 24, 36, and 72 hpi (magnification of 200×). (**b**) IFA results show the reactivity of a monoclonal antibody against PRRSV N protein to SCABTC-202305 and SCABTC-202309. (**c**) Electron micrographs of SCABTC-202305 and SCABTC-202309 particles. All bars are 100 nm. (**d**) Viral titers at 24, 36, and 72 hpi. (**e**) The growth curve of SCABTC-202305 and SCABTC-202309. Significant differences are marked with asterisks and “ns”: ****P < 0.0001; ***P < 0.001; **P < 0.01; *P < 0.05.

Serum samples positive for PRRSV were inoculated into Marc-145 cells under favorable growth conditions for virus isolation and identification. PRRSV was successfully isolated from the serum samples. The isolates showed increased CPE with increasing incubation. At 24 hours post-infection (hpi), minimal CPE was observed by aggregation of a few cells. At 48 hpi, the cells had aggregated, shrunk, and rounded significantly. At 72 hpi, numerous cells had clumped or detached (Fig. 1a). Typical CPE persisted after five passages, with viral nucleic acid levels in the culture medium gradually increasing with each passage. The IFA showed that both SCABTC-202305 and SCABTC202309 groups exhibited significant green fluorescence in contrast to the mock group, which showed no fluorescence (Fig. 1b). Viral particles of isolated strains can be visualized by projection electron microscopy (Fig. 1c). Virus titers are shown in Figure 1d and e. In this study, two PRRSV isolates, designated SCABTC-202305 and SCABTC-202309, were isolated and identified.

### Genome sequence and phylogenetic analysis

**Fig 2:**
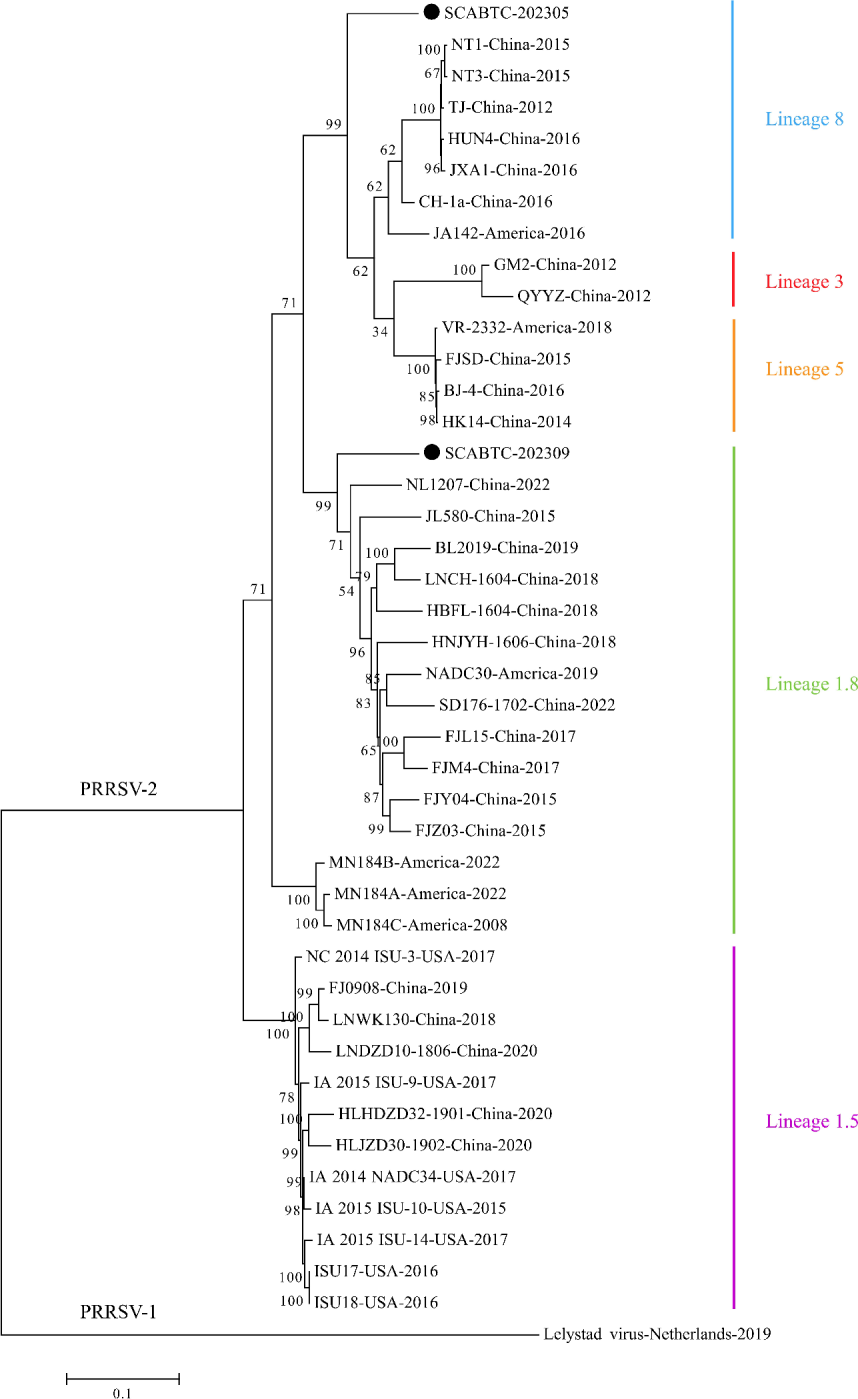
Phylogenetic tree based on the PRRSV complete gene sequence. The phylogenetic tree was constructed by the maximum likelihood method in MEGA7.0. ITOL version 6.9 (Biobyte, Heidelberg, Germany) was used to modify the genetic evolutionary tree, using different colors to distinguish different lineages and reference strains with GenBank sequence numbers. The circles (●) indicate the isolated strain in this study.

WGS of strains SCABTC-202305 and SCABTC-202309 showed their full lengths to be 15,017 and 14,993 bp, respectively. The genome sequences were deposited in GenBank under accession numbers OR365672 and OR766560. Phylogenetic analysis based on the WGS showed SCABTC-202305 clustered within lineage 8 and SCABTC-202309 clustered within lineage 1.8 (Fig. 2). Similarity analysis revealed SCABTC-202305 and SCABTC-202309 shared 88.7%/84.9%, 85.2%/84.4%, 85.2%/84.5%, and 91.6%/89.3% similarity with JXA1 (lineage 8), VR-2332 (lineage 5), IA/2014/NADC34 (lineage 1.5), and NADC30 (lineage 1.8), respectively. The similarity with the Lelystad virus (PRRSV-1) was only 60.1%/59.9% (Table 2).

**TABLE 2:** Pairwise genomic identity of the whole genome between the identified strains and representative strains of PRRSV

Region	Nucleotide identity % (SCABTC-202305/SCABTC-202309)					
	VR-2332 (lineage 5)	JXA1 (lineage 8)	NADC30 (lineage 1.8)	IA/2014/NADC34 (lineage 1.5)	QYYZ (lineage 3)	LV (PRRSV-1)
5'UTR	91.5/92.6	96.3/99.4	89.9/92.1	90.9/92.0	93.7/94.9	60.1/60.2
ORF1a	84.3/82.2	87.7/82.1	85.7/87.1	80.1/80.4	80.2/79.3	55.5/55.1
Nsp1	85.5/83.6	89.6/85.2	86.7/86.7	79.7/81.4	83.7/81.1	55.2/54.3
Nsp2	76.1/76.3	73.2/75.2	89.5/84.1	76.0/74.8	71.5/72.7	48.3/49.2
Nsp3	90.4/84.2	98.4/82.2	82.2/90.7	82.9/85.1	82.2/80.9	59.7/59.9
Nsp4	90.0/84.8	99.0/84.0	84.5/90.7	85.9/82.2	84.3/83.1	61.2/60.5
Nsp5	86.1/87.1	92.9/85.9	84.5/89.8	82.5/84.1	80.2/80.4	64.0/62.3
Nsp6	91.7/93.8	95.8/89.6	93.8/95.8	87.2/89.4	93.8/91.7	68.8/68.8
Nsp7	87.4/85.7	91.9/89.7	80.2/85.1	79.9/81.3	89.2/86.4	53.0/52.5
Nsp8	94.1/94.8	96.3/97.0	87.4/86.7	88.9/88.1	94.0/92.5	65.2/64.4
ORF1b	90.3/86.4	96.4/86.9	86.2/90.8	85.6/87.7	90.3/85.0	63.3/63.5
Nsp9	90.4/87.2	92.0/88.5	85.5/90.2	85.8/87.7	91.2/85.7	66.6/67.0
Nsp10	90.1/84.8	99.8/84.3	84.0/91.9	85.5/90.4	90.6/84.6	60.2/61.2
Nsp11	90.9/87.0	99.7/87.3	89.9/89.7	86.9/85.2	89.0/84.3	65.8/65.9
Nsp12	90.0/87.4	100.0/87.6	89.6/91.7	83.5/84.6	87.4/84.8	51.1/50.0
ORF2	92.9/84.9	99.5/84.8	85.2/90.4	86.4/83.3	90.4/84.1	64.6/64.6
ORF3	89.4/81.5	92.8/79.4	82.8/87.7	82.8/82.0	82.9/82.0	64.6/64.8
ORF4	90.1/86.0	97.6/84.7	85.5/92.9	86.2/93.7	94.8/84.5	66.3/68.5
ORF5	87.1/84.3	97.3/83.6	84.2/92.0	85.4/87.6	82.1/83.4	63.9/62.3
ORF6	94.9/89.0	99.4/88.8	89.1/96.6	89.0/93.5	90.3/89.3	69.3/70.9
ORF7	91.7/89.2	97.0/87.5	89.2/90.6	87.5/90.3	90.3/88.1	65.3/65.9
3'UTR	90.7/91.2	90.9/89.7	88.4/97.9	86.7/95.9	87.3/91.2	73.5/74.3
Complete genome	85.2/84.4	88.7/84.9	91.6/89.3	85.2/84.5	82.7/82.8	60.1/59.9

We searched the similar strains of the isolates in this experiment using the NCBI database (Table 3). The results showed that SCABTC-202305 was highly similar to the strain SC/DJY (98.43%) detected in Chengdu, Sichuan Province, suggesting that it might be a novel mutant of the endemic strain SC/DJY. On the other hand, SCABTC-202309 was identified as a new recombinant strain reported in Zunyi, Guizhou Province. The most similar strain, SDYG1606, exhibited only 90.94% similarity.

**TABLE 3:** Information on the related recombinant strains of the isolates

Isolates	Related strains	Parent strain major/minor	Recombination regions	Similarity	Location	Accession no.
SCABTC- 202305	SC/DJY	NADC30/JXA1	Nsp9, ORF2-3	98.43%	Sichuan	MT075480.1
	CH/SCCD-2/2018	NADC30/JXA1	Nsp4-7	90.54%	Sichuan	MZ747437.1
	FJWQ16	NADC30/JXA1	Nsp7-9	90.69%	Fujian	KX758249.1
SCABTC- 202309	SDYG1606	JXA1/NADC30	Nsp3-9	90.94%	Shandong	KY053458.1
	15SC3	NADC30/JXA1	Nsp1-2, Nsp9	90.75%	Sichuan	KX815428.1
	15LN3	NADC30/JXA1	Nsp4-9	90.20%	Liaoning	KX815425.1

### Amino acid mutation analysis

Previous studies have shown that among the different proteins of PRRSV, GP5 and NSP2 have the lowest amino acid similarity, suggesting that the GP5 and NSP2 proteins are most susceptible to mutation (30). Therefore, they are usually used as targets for epidemiological analysis (31).

**Fig 3:**
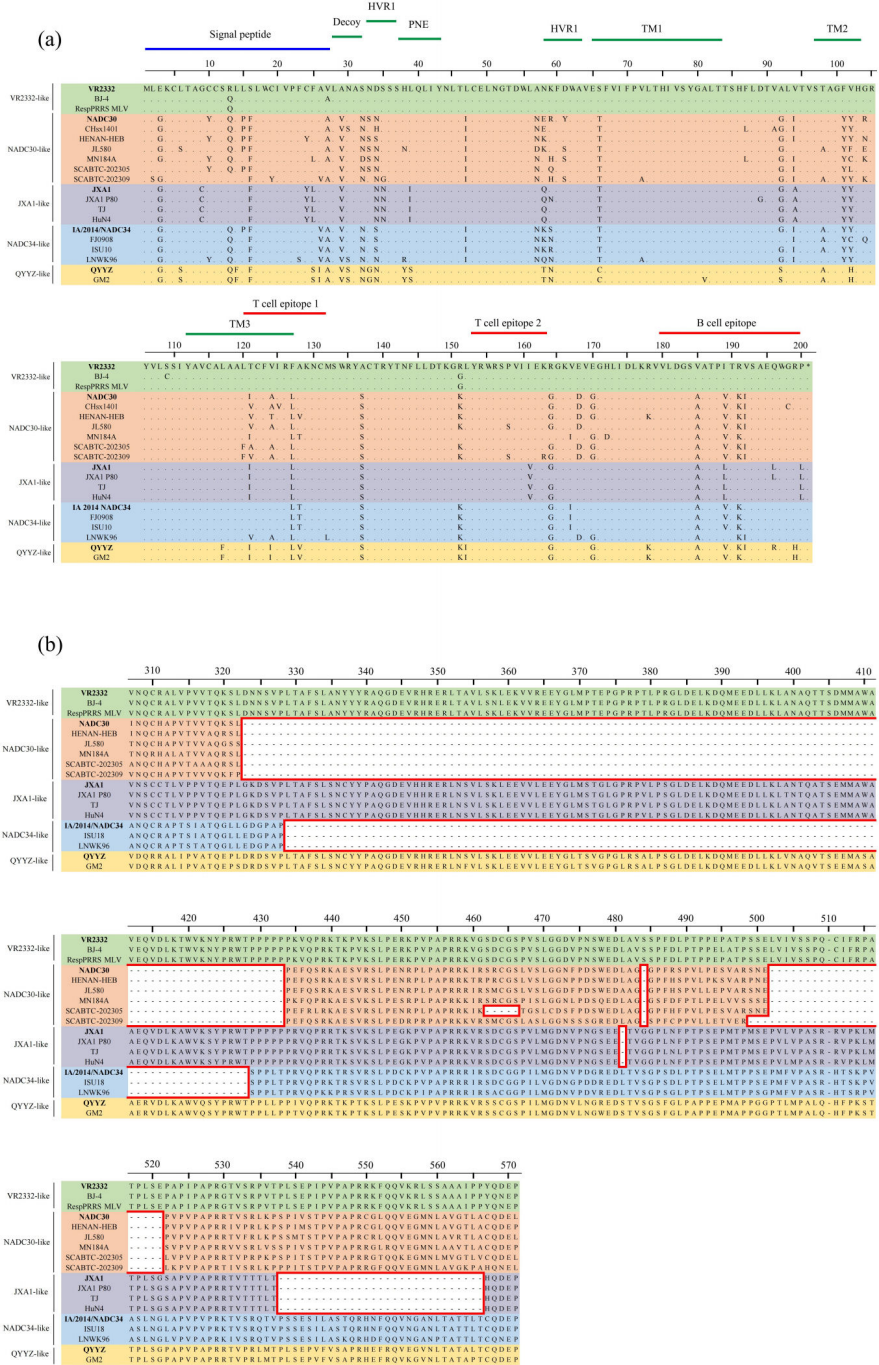
Amino acid sequence alignment of the ORF5 and Nsp2 HVR. (**a**) Multiple alignments of GP5 amino acid sequences of SCABTC-202305 and SCABTC-202309 and 18 PRRSV reference strains. (**b**) Multiple alignments of Nsp2 HVR amino acid sequences of SCABTC-202305 and SCABTC-202309 and 18 PRRSV reference strains. Deleted amino acids are indicated in red boxes, with different colors distinguishing the various PRRSV lineages.

The major envelope protein GP5, encoded by ORF5, is one of the most variable PRRSV proteins (15). Results with other representative strains show a wide range of amino acid substitutions in the signal peptide, hypervariable regions 1 and 2 (HVR1 and HVR2), and potential neutralizing epitopes (PNE) of ORF5. Several amino acid substitutions, including S^16^→F^16^, A^29^→V^29^, S^32^→N^32^, D^34^→N^34^, L^47^→I^47^, A^57^→N^57^, S^66^→T^66^, A^92^→G^92^, V^94^→I^94^, F^101^→Y^101^, V^124^→A^124^, F^127^→L^127^, A^137^→S^137^, R^151^→K^151^, R^164^→G^164^, E^168^→D^168^, E^170^→G^170^, V^185^→A^185^, I^189^→V^189^, R^191^→K^191^, and V^192^→I^192^, were found in NADC30-like strains, including SCABTC-202305 and SCABTC-202309. Notably, the isolates in this study contained several unique amino acid substitutions in the ORF5 gene. Specifically, SCABTC-202305 underwent the amino acid substitutions K^59^→Q^59^, V^102^→L^102^, and T^121^→A^121^, while SCABTC-202309 had the substitutions L^2^→S^2^, C^19^→Y^19^, A^26^→V^26^, S^35^→G^35^, V^72^→A^72^, and K^163^→R^163^ (Fig. 3a).

Nsp2 is one of the most variable proteins of PRRSV and contains different patterns of deletions and insertions compared to the prototype strain VR2332 (32). In this study, isolates SCABTC-202305 and SCABTC-202309 exhibited a discontinuous deletion pattern of 131 amino acids (111 + 1 + 19) characteristic of NADC30-like strains compared to the prototype PRRSV-2 strain VR2332. Interestingly, SCABTC-202305 contained an additional five amino acid deletion in Nsp2 from positions 462-466, while SCABTC-202309 had an additional three amino acid deletion in Nsp2 from positions 499-501 (Fig. 3b).

### Recombination analysis

**Fig 4:**
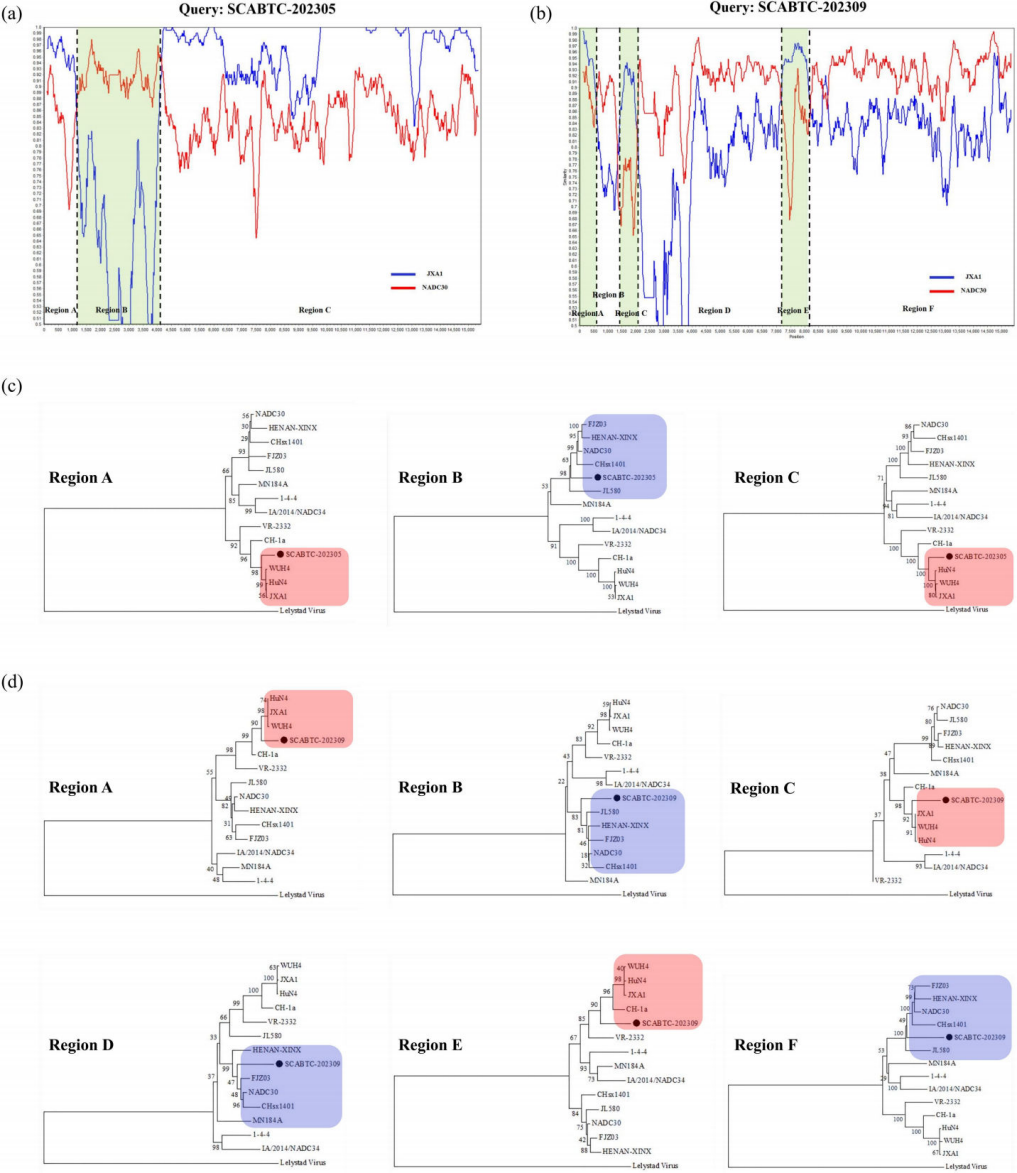
Genome recombination analysis of the PRRSV isolates SCABTC-202305 and SCABTC-202309. Recombination analysis was calculated using SimPlot 3.5.1 software. (**a**) SCABTC-202305 (query) with JXA1 (red) and NADC30 (blue). (**b**) SCABTC-202309 (query) with JXA1 (red) and NADC30 (blue). Recombination breakpoints are shown with black dotted lines, and the locations are shown at the bottom. The green regions are the minor parental regions, whereas the white regions are the major parental regions. (**c**) The phylogenetic tree was based on each recombinant region of SCABTC-202305. The red area represents the major parental regions, and the blue area represents the minor parental regions. (**d**) The phylogenetic tree was based on each recombinant region of SCABTC-202309. The red area represents the major parental regions, and the blue area represents the minor parental regions.

To identify possible recombination events in the isolated strains, we performed recombination analyses using SimPlot and RDP software. The results revealed that both SCABTC-202305 and SCABTC-202309 strains isolated in this study underwent genetic recombination. SCABTC-202305 was a recombinant of JXA1 and NADC30, with one recombination event identified (Fig. 4a). The major parental strain was JXA1, and the minor parental strain was NADC30. Recombination event occurred at nucleic acids 1110-4089 nt (Region B). Phylogenetic analyses were conducted on the whole genome and the recombination regions. The phylogenetic tree showed that the major region of SCABTC-202305 belongs to lineage 8, while the recombination region belongs to lineage 1.8 (Fig. 4c). SCABTC-202309 was a recombinant of NADC30 and JXA1, with three recombination events identified. The major parental strain was NADC30, and the minor parental strain was JXA1 (Fig. 4b). Recombination event 1 occurred at nucleic acids 1-561 nt. Recombination event 2 occurred at nucleic acids 1321-2041 nt. Recombination event 3 occurred at nucleic acids 7161-8201 nt. Phylogenetic analyses were performed on the whole genome and the recombination regions. The genome-wide phylogenetic tree showed that SCABTC-202309 belongs to lineage 1.8, while all recombination regions belong to lineage 8 (Fig. 4d).

### Clinical signs

**Fig 5:**
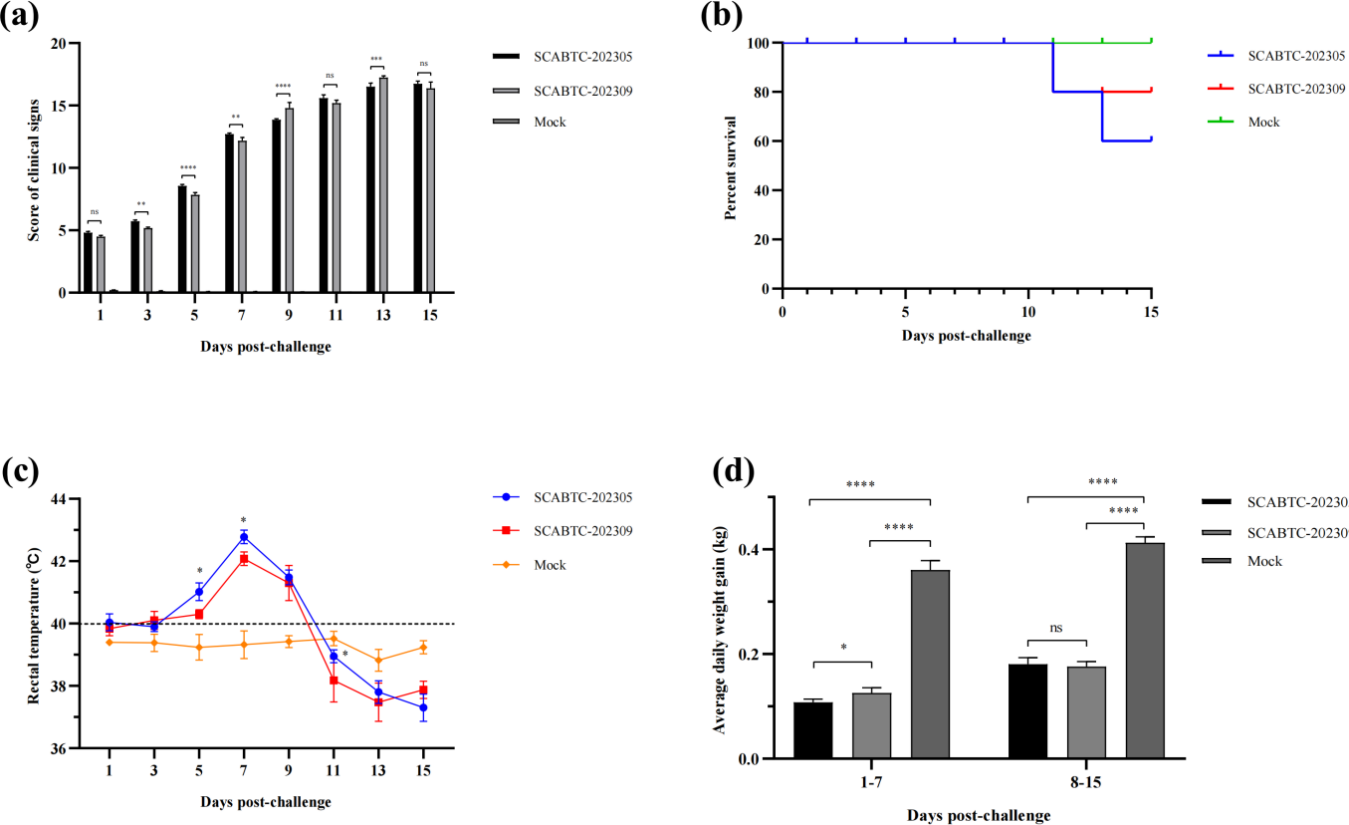
Pathogenicity results of piglets. (a) Clinical scores, (b) survival rate, (c) rectal temperature changes, and (d) body weight change of piglets in each group. Significant differences are marked with asterisks and “ns”: ****P < 0.0001; ***P < 0.001; **P < 0.01; *P < 0.05.

The pathogenicity of the SCABTC-202305 and SCABTC-202309 strains was compared in 1-week-old piglets. All PRRSV-infected piglets exhibited severe respiratory impairment (Fig. 5a). In particular, clinical signs were more pronounced in piglets infected with SCABTC-202305 than in those infected with SCABTC-202309. Control piglets showed no significant clinical signs. Piglets infected with SCABTC-202305 showed a febrile response from 1 dpi, with rectal temperatures remaining above 40°C from 3 to 9 dpi and peaking at 42.9°C at 7 dpi (Fig. 5c). Clinical signs such as coughing, sneezing, and anorexia began at 3 dpi, and severe respiratory symptoms, including dyspnea, tachypnea, and shivering, started at 7 dpi. One piglet died at 11 dpi, and one at 13 dpi (Fig. 5b). Piglets infected with SCABTC-202309 had a febrile response only from 3 dpi, with temperatures above 40°C from 5 to 9 dpi and peaking at 42.4°C at 7 dpi (Fig. 5c). Coughing, sneezing, and anorexia appeared at 3 dpi, and two piglets showed dyspnea and tachypnea at 7 dpi. One piglet died at 11 dpi (Fig. 5b). Furthermore, the average daily weight gain of piglets infected with SCABTC-202305 and SCABTC-202309 was 0.108 and 0.126 kg, respectively, at 1–7 dpi, and 0.181 and 0.176 kg, respectively, at 8–15 dpi (Fig. 5d). The growth performance of PRRSV-infected piglets was significantly weaker than that of control piglets throughout the experiment. At 1–7 dpi, piglets infected with SCABTC-202305 gained significantly less weight than those infected with SCABTC-202309.

### Viremia and viral excretion

**Fig 6:**
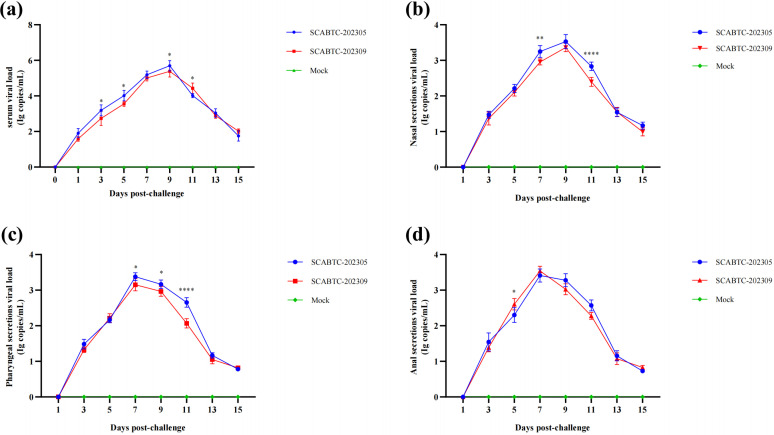
Viral load in (a) the blood, (b) nasal secretions, (c) pharyngeal secretions, and (d) anal secretions. Significant differences are marked with asterisks and “ns”: ****P < 0.0001; **P < 0.01; *P < 0.05.

Serum, nasal swabs, pharyngeal swabs, and anal swabs were collected from pigs at 1, 3, 5, 7, 9, 11, 13, and 15 dpi to evaluate viremia and viral shedding. Both PRRSV isolates SCABTC-202305 and SCABTC-202309 were detected in serum starting at 1 dpi, with peak viral titers observed at 9 dpi. Following the peak, serum viral titers decreased slightly. Specifically, SCABTC-202305 inoculation resulted in significantly higher serum viral RNA levels at 3, 5, and 9 dpi, but lower levels at 11 dpi compared to SCABTC-202309. No viremia was detected in the control groups at any point in time (Fig. 6a). Both PRRSV strains were detected in nasal, pharyngeal, and anal swabs starting at 3 dpi, with peak viral shedding occurring at 7–9 dpi. Respiratory viral shedding was significantly higher in SCABTC-202305-infected pigs compared to SCABTC-202309 at 7 and 11 dpi. No significant difference in gastrointestinal shedding was observed between the two PRRSV strains (Fig. 6b through d).

### Tissue tropism analysis

**Fig 7:**
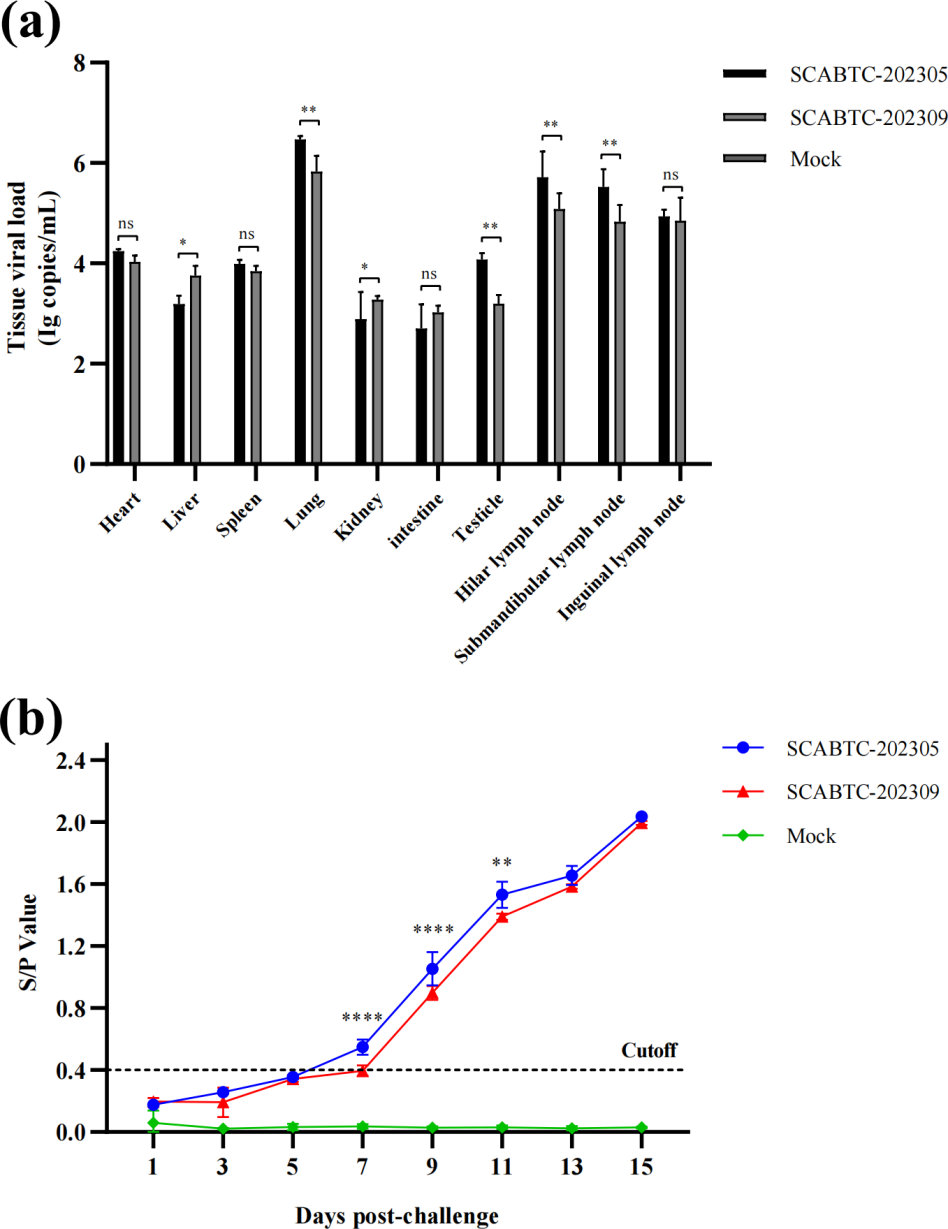
(**a**) Viral load in the heart, liver, spleen, lung, kidney, intestine, testicle, hilar lymph node, submandibular lymph node, and inguinal lymph node of piglets. (**b**) Detection of PRRSV-specific antibody in piglets of each group. Significant differences are marked with asterisks and “ns”: ****P < 0.0001; **P < 0.01; *P < 0.05.

The viral load of different tissues in piglets after the PRRSV attack was determined by qRT-PCR (Fig. 7a). The viral RNA was not detected in any of the samples from the mock group. The tissues with the highest viral load were the lungs and lymph nodes, followed by the heart, spleen, and testes. The liver, kidney, and intestines had the lowest viral load. The SCABTC-202305 group had a significantly higher viral load in the lungs, testes, hilar lymph nodes, and submandibular lymph nodes than the SCABTC-202309 group. The SCABTC-202309 group had a significantly lower viral load in the liver and kidney than the SCABTC-202305 group. There was no significant difference in the viral load of the spleen, intestine, and inguinal lymph nodes between the two groups.

### Humoral immune response

After PRRSV infection, pig sera were collected at 1, 3, 5, 7, 9, 11, 13, and 15 dpi to detect PRRSV IgG antibodies. Analysis of PRRSV antibody levels showed seroconversion in all piglets of both infected groups at 7 dpi, with antibody titers continuing to increase until plateauing at 15 dpi. Antibody titers were significantly higher in pigs infected with SCABTC-202305 compared to those infected with SCABTC-202309 at 7, 9, and 11 dpi. At 1, 3, 5, 13, and 15 dpi, no significant difference in antibody titers was observed between the two infected groups. The control group remained negative for PRRSV-specific antibodies throughout the experiment (Fig. 7b).

### Clinical anatomical, histopathology, and immunohistochemical analysis

**Fig 8:**
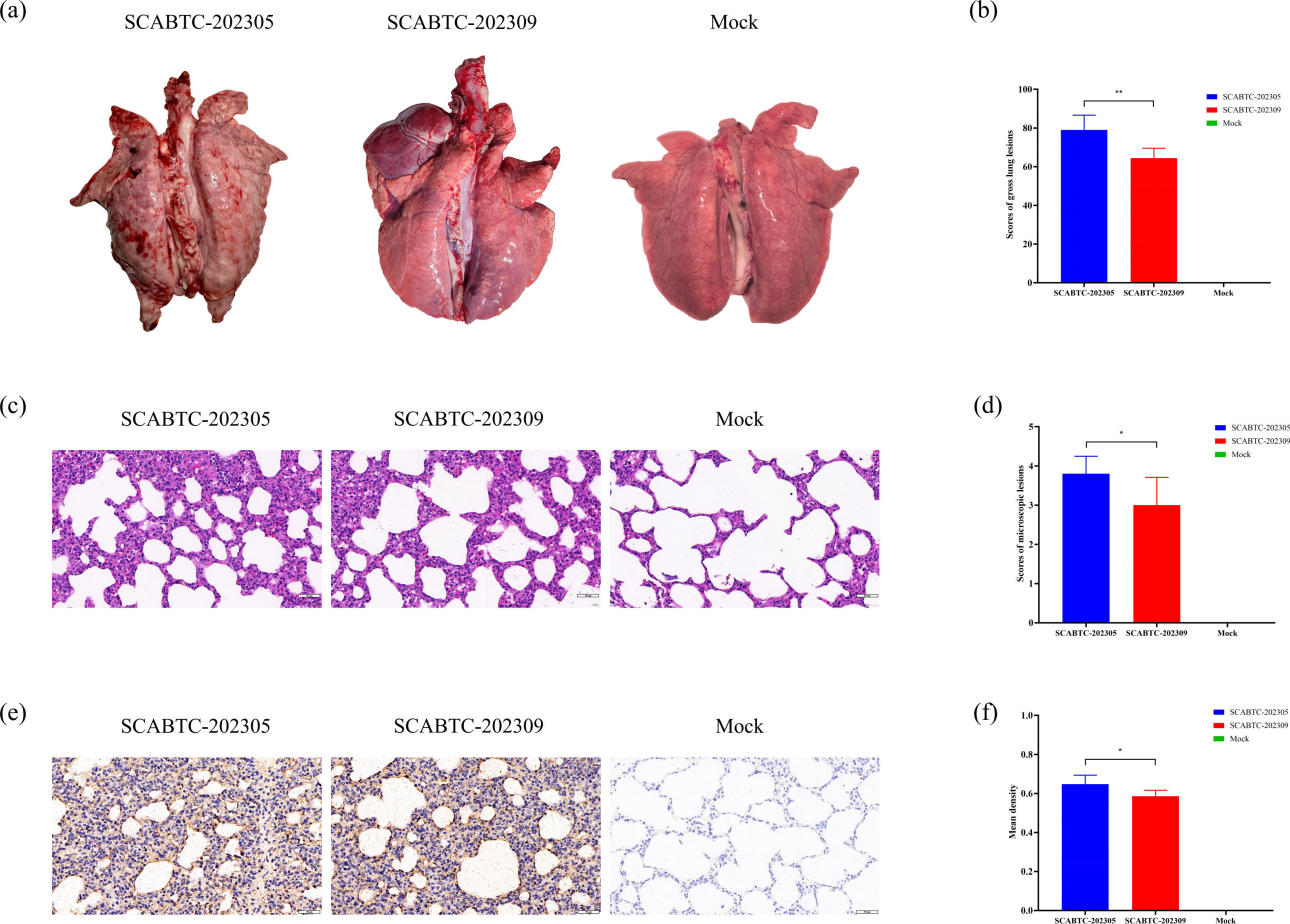
(**a**) Gross lesions in piglet lungs. Piglets inoculated with SCABTC-202305 developed severe interstitial pneumonia. Piglets inoculated with SCABTC-202309 exhibited moderate interstitial pneumonitis with consolidation. Piglets in the mock group showed no gross lung lesions. (**b**) Scores of lung gross examination of the inoculated piglets. The lung gross lesions were scored based on the percentage of lung area affected. (**c**) Microscopic lesions of the lung of piglets. Piglets inoculated with SCABTC-202305 exhibited thickened alveolar septa, alveolar epithelial cell degeneration, and inflammation (magnification of 400×). Piglets inoculated with SCABTC-202309 exhibited thickened alveolar septa (magnification of 400×). Piglets in the mock group had no evident lung pathology (magnification of 400×). (**d**) Scores of lung microscopic lesions examination of the inoculated piglets. The microscopic lung lesions were scored based on the extent and severity of interstitial pneumonia. (**e**) Detection of PRRSV in lung tissues of piglets by IHC (magnification of 400×). (**f**) The mean density of lung IHC staining sections of inoculated piglets. Mean density is calculated by dividing the total optical density of the positive signals in each field of view by the area of the effective target distribution.

At necropsy, PRRSV infection mainly caused lung consolidation and interstitial pneumonia in piglets (33). Lung lesions in piglets infected with SCABTC-202305 were severe, with diffuse interstitial pneumonia, lung swelling, hard, rubbery texture, and diffuse bleeding at the edge of the lung. The lungs of piglets infected with SCABTC-202309 showed mild interstitial pneumonia and pulmonary edema (Fig. 8a and b). Microscopic histopathological examination also showed that piglets infected with PRRSV developed marked thickening of the alveolar septum, necrosis of alveolar epithelial cells, and an increase in inflammatory cells, suggesting the development of interstitial pneumonia (Fig. 8c and d). The results showed that the gross and microscopic lung lesions of piglets infected with SCABTC-202305 were more significant than those of piglets infected with SCABTC-202309, while no obvious lesions were observed in the lungs of piglets in the control group. The distribution of PRRSV in the lungs was studied using IHC staining. No positive signals were detected in the control group, whereas PRRSV-positive signals were observed in the infected group. These signals appeared brown and were distributed in alveolar cells and macrophages, etc. (Fig. 8e and f). The mean optical density of the positive signals was significantly higher in SCABTC-202305-infected piglets compared to SCABTC-202309 (*P* < 0.05).

## DISCUSSION

Since 2006, the emergence of lineage 8 (HP-PRRSV strains) has caused significant harm to the Chinese pig industry. After 2010, the MLV has been widely used to control the disease (6, 34). However, the high mutation and recombination rates of PRRSV have led to its recurrence, presenting challenges in containing PRRSV (14, 35). After 2013, lineage 1.8 (NADC30-like strains) emerged and spread rapidly and widely. Currently, the NADC30-like strain is prevalent in multiple provinces in China, causing an abortion storm and may have become the main epidemic strain of PRRSV (30). Our findings are consistent with the epidemiological trend. In this study, two novel PRRSV isolates, SCABTC-202305 and SCABTC-202309, were isolated from different regions of Southwest China. Phylogenetic analysis based on the complete genome sequences revealed that SCABTC-202305 belongs to lineage 8, and SCABTC-202309 belongs to lineage 1.8.

To determine the prevalence of the isolated strains across various geographical regions, we searched the NCBI database for strains that are similar to the isolates in this study. SCABTC-202305 appears to be a novel variant of the SC/DJY strain, previously reported in Chengdu, Sichuan Province, in 2020 (31). This strain remains predominantly confined to Sichuan Province, and no cases have been reported in other regions. It is worth noting that Sichuan Province serves as the primary center for pig breeding in Southwest China (36). This situation potentially contributes to the rapid cross-regional transmission of the strain through frequent mutations and recombinations (37). Therefore, it is vital to continuously monitor the prevalence of SCABTC-202305 over an extended period and develop an associated vaccine. In contrast, SCABTC-202309 represents a novel recombinant strain, exhibiting only 90.94% similarity to its closest strain, SDYG1606 (38). This significant differentiation is attributed to a distinct and intricate recombination pattern involving three recombination events. The emergence of this novel recombination pattern raises concerns regarding the epidemiology of PRRSV and necessitates ongoing monitoring of potential risks.

Mutation and deletions are important mechanisms in PRRSV evolution (27). In China, PRRSV-2 isolates were categorized into 1, 3, 5, and 8 lineages based on variation in the NSP2 gene (37). HP-PRRSV typically has a discontinuous 30 amino acid (1 + 29) deletion, while NADC30-like strains have a 131 amino acid (111 + 1 + 19) deletion (39). In this study, SCABTC-202305 had an additional five amino acid deletion in Nsp2 from position 462-466, and SCABTC-202309 had a three amino acid deletion from position 499-501, relative to other NADC30-like PRRSV. Nsp2 contains several immunogenic epitopes, indicating that it is an immunogenic protein in PRRSV that triggers specific antibody production during infection (40). These additional Nsp2 deletions may affect virulence and increase viral fitness in the host, favoring the occurrence of immune escape (41). Additionally, these isolates had unique mutations in ORF5 that may alter antigenicity (42). SCABTC-202305 had K^59^→Q^59^, V^102^→L^102^, and T^121^→A^121^ changes in GP5, while SCABTC-202309 had L^2^→S^2^, C^19^→Y^19^, A^26^→V^26^, N^30^→S^30^, S^35^→G^35^, V^72^→A^72^, and K^163^→R^163^ substitutions. This suggests that the virus has undergone specific mutations at certain sites during its ongoing mutation. This may make it more virulent than other viruses (15). As GP5 mediates receptor binding and neutralizing antibodies, these mutations likely change antigenic properties and host–cell interactions (30). This may be a significant factor contributing to the incomplete immune protection provided by existing vaccines for pigs. In addition, previous studies have shown that the amino acid at position 137 of the GP5 protein distinguishes between attenuated vaccine strains (A) and wild strains (S) (43). The sequencing results indicate that both isolates are wild strains.

Frequent recombination of PRRSV strains is an important evolutionary mechanism that allows the virus to rapidly adapt and evade host immunity (35). This poses a significant challenge to the prevention and control of PRRSV. The NADC30-like PRRSV strains may be particularly susceptible to recombination due to highly variable genomic regions, leading to genetic instability (13). Numerous studies in China have reported recombination between NADC30-like (lineage 1.5), HP-PRRSV (lineage 8), and C-PRRSV (lineage 5) strains (44, 45). Such recombination events are thought to have contributed to the epidemic spread of NADC30-like PRRSV since 2013 (46). Concerningly, some virulent strains such as TJnh1501, FJXS15, and 15HEN1 appear to be natural recombinants between HP-PRRSV vaccine strains and NADC30-like field strains (47–49). Overall, the propensity of PRRSV strains to recombine is an ongoing challenge to disease control that warrants further study and vigilance.

In animal experiments, SCABTC-202305 and SCABTC-202309 exhibited moderate virulence between the JXA1 and NADC30 strains. Recombination analyses demonstrated that both isolates underwent recombination events, but albeit with different recombination patterns. SCABTC-202305 was a recombinant between JXA1 and NADC30, with JXA1 being the major parent strain. One recombination event was identified in the SCABTC-202305 strain. It occurred in the Nsp1 and Nsp2 gene regions (1110-4089 nt). Recombination events in the nonstructural proteins, particularly Nsp2, likely altered the pathogenicity and replication ability compared to the major and minor PRRSV parent strains for the strain (36, 38, 47). Because Nsp2 is an important immunogenic protein involved in viral replication and pathogenesis (50), the recombination in this region with the NADC30 strain as the major parent likely conferred increased replication capacity compared to the JXA1 parent strain (32). Furthermore, the recombination of Nsp1 may also have impacted the ability of transcription and replication, as it encodes the regulatory protein responsible and inhibits the secretion of interferon (51). SCABTC-202309 was a recombinant between NADC30 and JXA1, with NADC30 being the major parent strain. There are three recombination events that occurred in the SCABTC-202309 strain. One in the 5' UTR (1-561 nt), another in the gene region containing partial Nsp1 and Nsp2 (1321-2041 nt), and third in the gene region containing Nsp8 and Nsp9 (7161-8201 nt). The recombination events in the Nsp1 and Nsp2 regions with the JXA1 minor parent may have increased virulence compared to the NADC30 major parent. However, because the recombination was limited to a partial area, it may only have a modest effect on the virulence of the strain. In addition, the Nsp8 and Nsp9 recombination could potentially have affected viral replication (52).

Notably, SCABTC-202305 exhibits greater virulence and replication proficiency compared to SCABTC-202309, chiefly attributed to its predominant parental derivation from the highly pathogenic strain JXA1. It is worth noting that recent evidence has revealed that alterations in the genetic information of specific genes in the genome may directly affect the pathogenicity of novel PRRSV strains (53). PRRSV recombination can sometimes generate strains with enhanced virulence, depending on the genomic regions exchanged and parental viruses involved (6). Studies have shown that the recombination of NADC30-like PRRSV with other strains can increase pathogenicity, as evidenced by recombinant strains such as GZgy17 and SD-YL1712 (40, 54). The regions encoding the major structural proteins GP5, M, and N in SCABTC-202305 originate from the JXA1 strain. In contrast, the structural proteins in SCABTC-202309 were derived from the NADC30 strain, which may result in greater decay of infectivity and virulence. Furthermore, the region derived from the JXA1 strain in SCABTC-202305 encodes minor structural proteins GP2, GP3, and GP4 implicated in immunomodulation through receptor interactions (35). Alterations in these proteins may enable SCABTC-202305 to suppress or circumvent host immunity more potently than SCABTC-202309. Critically, nonstructural proteins such as Nsp8, Nsp9, and Nsp10 involved in genomic replication and transcription originate from the JXA1 parent in SCABTC-202305, preserving the maximal replication proficiency of JXA1, unlike the origins of these proteins in SCABTC-202309.

In summary, two novel PRRSV strains with distinct recombination patterns, designated SCABTC-202305 and SCABTC-202309, were isolated from piglet sera collected in Southwest China. Phylogenetic analyses demonstrated that SCABTC-202305 clustered within lineage 8, and SCABTC-202309 clustered within lineage 1.8. Additionally, multiple novel amino acid and nucleotide deletions were identified in the ORF5 and Nsp2 genes. Animal studies in piglets showed both isolates were highly pathogenic, with SCABTC-202305 exhibiting greater virulence and replication capacity compared to SCABTC-202309. The results of recombination analysis showed that the virulence genes of PRRSV isolates were changed due to recombination, which affected the pathogenicity of the strains. This finding provides a reference for the surveillance and control of the PRRSV epidemic in Southwest China.

## Data Availability

The data that support the findings of this study are available from the corresponding author on reasonable request. The PRRSV-sequences are available in GenBank, accession number OR365672 and OR766560.

## SUPPLEMENTAL MATERIAL

Table S1 spectrum.04071-23-s0001.doc The reference sequence information of PRRSV.

ASM does not own the copyrights to Supplemental Material that may be linked to, or accessed through, an article. The authors have granted ASM a non-exclusive, world-wide license to publish the Supplemental Material files. Please contact the corresponding author directly for reuse.

## References

[1] Shi M, Lam TT-Y, Hon C-C, Hui RK-H, Faaberg KS, Wennblom T, Murtaugh MP, Stadejek T, Leung FC-C. 2010. Molecular epidemiology of PRRSV: a phylogenetic perspective. Virus Res 154:7–17. doi:10.1016/j.virusres.2010.08.014 20837072

[2] Kappes MA, Faaberg KS. 2015. PRRSV structure, replication and recombination: origin of phenotype and genotype diversity. Virology 479–480:475–486. doi:10.1016/j.virol.2015.02.012 PMC7111637 25759097

[3] Dokland T. 2010. The structural biology of PRRSV. Virus Res 154:86–97. doi:10.1016/j.virusres.2010.07.029 20692304 PMC7114433

[4] Nguyen NH, Tran HAT, Nguyen TQ, Nguyen PBT, Le THT, Lai DC, Nguyen MN. 2022. Phylogenetic analysis of porcine reproductive and respiratory syndrome virus in Vietnam, 2021. Virus Genes 58:361–366. doi:10.1007/s11262-022-01912-w 35589912 PMC9119219

[5] Sun Q, Xu H, An T, Cai X, Tian Z, Zhang H. 2023. Recent progress in studies of porcine reproductive and respiratory syndrome virus 1 in China. Viruses 15:1528. doi:10.3390/v15071528 37515213 PMC10384046

[6] Guo Z, Chen X, Li R, Qiao S, Zhang G. 2018. The prevalent status and genetic diversity of porcine reproductive and respiratory syndrome virus in China: a molecular epidemiological perspective. Virol J 15:2. doi:10.1186/s12985-017-0910-6 29301547 PMC5753475

[7] Zhang H, Leng C, Ding Y, Zhai H, Li Z, Xiang L, Zhang W, Liu C, Li M, Chen J, Bai Y, Kan Y, Yao L, Peng J, Wang Q, Tang YD, An T, Cai X, Tian Z, Tong G. 2019. Characterization of newly emerged NADC30-like strains of porcine reproductive and respiratory syndrome virus in China. Arch Virol 164:401–411. doi:10.1007/s00705-018-4080-7 30353281

[8] Wang X, Zhang K, Mo Q, Chen G, Lv J, Huang J, Pang Y, Wang H, Liu W, Huang K, Min X, Ren T, Ouyang K, Chen Y, Huang W, Wei Z. 2022. The emergence and pathogenesis of recombinant viruses associated with NADC34-like strains and the predominant circulating strains of porcine reproductive and respiratory syndrome virus in southern China. Viruses 14:1695. doi:10.3390/v14081695 36016319 PMC9416154

[9] Montaner-Tarbes S, Del Portillo HA, Montoya M, Fraile L. 2019. Key gaps in the knowledge of the porcine respiratory reproductive syndrome virus (PRRSV). Front Vet Sci 6:38. doi:10.3389/fvets.2019.00038 30842948 PMC6391865

[10] Xu H, Gong B, Sun Q, Li C, Zhao J, Xiang L, Li W, Guo Z, Tang Y, Leng C, Li Z, Wang Q, Zhou G, An T, Cai X, Tian Z-J, Peng J, Zhang H. 2023. Genomic characterization and pathogenicity of BJEU06-1-like PRRSV-1 ZD-1 isolated in China. Transbound Emerg Dis 2023:1–12. doi:10.1155/2023/6793604 PMC12016759 40303662

[11] Jiang Y, Li G, Yu L, Li L, Zhang Y, Zhou Y, Tong W, Liu C, Gao F, Tong G. 2020. Genetic diversity of porcine reproductive and respiratory syndrome virus (PRRSV) from 1996 to 2017 in China. Front Microbiol 11:618. doi:10.3389/fmicb.2020.00618 32390968 PMC7193098

[12] Chang H, Zheng J, Qiu Y, Chen C, Li Q, Wu Q, Lin L, Zhao H, Zhou Q, Gong L, Sun Y, Zhang X, Wang H. 2023. Isolation, identification, and pathogenicity of a NADC30-like porcine reproductive and respiratory disorder syndrome virus strain affecting sow production. Front Vet Sci 10:1207189. doi:10.3389/fvets.2023.1207189 37483283 PMC10360194

[13] Yu Y, Zhang Q, Cao Z, Tang YD, Xia D, Wang G, Shan H. 2021. Recent advances in porcine reproductive and respiratory syndrome virus NADC30-like research in China: molecular characterization, pathogenicity, and control. Front Microbiol 12:791313. doi:10.3389/fmicb.2021.791313 35087492 PMC8787316

[14] Wu Y, Peng O, Xu Q, Li Q, Li W, Lin L, Zhou Q, Cai X, Hu G, He Z, Chen Y, Zhang H. 2022. Characterization and pathogenicity of two novel PRRSVs recombined by NADC30-like and NADC34-like strains in China. Viruses 14:2174. doi:10.3390/v14102174 36298730 PMC9607012

[15] Huang J. 2017. Preparation of synthetic peptide antibody against GP5 protein of PRRSV SC strain and its ADE effect. Master. Sichuan agricultural university

[16] Kumar S, Stecher G, Tamura K. 2016. MEGA7: molecular evolutionary genetics analysis version 7.0 for bigger datasets. Mol Biol Evol 33:1870–1874. doi:10.1093/molbev/msw054 27004904 PMC8210823

[17] Chen XW, Li L, Yin M, Wang Q, Luo WT, Ma Y, Pu ZH, Zhou JL. 2017. Cloning and molecular characterization of the ORF5 gene from a PRRSV-SN strain from southwest China. Microb Pathog 112:295–302. doi:10.1016/j.micpath.2017.09.011 28970171

[18] Clewley JP. 1995. Macintosh sequence analysis software. DNAStar's LaserGene. Mol Biotechnol 3:221–224. doi:10.1007/BF02789332 7552691

[19] Martin D, Rybicki E. 2000. RDP: detection of recombination amongst aligned sequences. Bioinformatics 16:562–563. doi:10.1093/bioinformatics/16.6.562 10980155

[20] Padidam M, Sawyer S, Fauquet CM. 1999. Possible emergence of new geminiviruses by frequent recombination. Virology 265:218–225. doi:10.1006/viro.1999.0056 10600594

[21] Martin DP, Posada D, Crandall KA, Williamson C. 2005. A modified bootscan algorithm for automated identification of recombinant sequences and recombination breakpoints. AIDS Res Hum Retroviruses 21:98–102. doi:10.1089/aid.2005.21.98 15665649

[22] Smith JM. 1992. Analyzing the mosaic structure of genes. J Mol Evol 34:126–129. doi:10.1007/BF00182389 1556748

[23] Posada D, Crandall KA. 2001. Evaluation of methods for detecting recombination from DNA sequences: computer simulations. Proc Natl Acad Sci U S A 98:13757–13762. doi:10.1073/pnas.241370698 11717435 PMC61114

[24] Gibbs MJ, Armstrong JS, Gibbs AJ. 2000. Sister-scanning: a Monte Carlo procedure for assessing signals in recombinant sequences. Bioinformatics 16:573–582. doi:10.1093/bioinformatics/16.7.573 11038328

[25] Lam HM, Ratmann O, Boni MF. 2018. Improved algorithmic complexity for the 3SEQ recombination detection algorithm. Mol Biol Evol 35:247–251. doi:10.1093/molbev/msx263 29029186 PMC5850291

[26] Murtaugh MP, Yuan S, Faaberg KS. 2001. Appearance of novel PRRSV isolates by recombination in the natural environment. Adv Exp Med Biol 494:31–36. doi:10.1007/978-1-4615-1325-4_4 11774486

[27] Xia Y, Zhang T, Gong D, Qi J, Jiang S, Yang H, Zhu X, Gan Y, Zhang Y, Han Y, Li Y, Li J. 2023. Recombination and mutation in a new HP-PRRSV strain (SD2020) from China. Viruses 15:165. doi:10.3390/v15010165 36680205 PMC9864264

[28] Li Y, Zhou L, Zhang J, Ge X, Zhou R, Zheng H, Geng G, Guo X, Yang H. 2014. Nsp9 and Nsp10 contribute to the fatal virulence of highly pathogenic porcine reproductive and respiratory syndrome virus emerging in China. PLOS Pathog. 10:e1004216. doi:10.1371/journal.ppat.1004216 24992286 PMC4081738

[29] Halbur PG, Paul PS, Meng X-J, Lum MA, Andrews JJ, Rathje JA. 1996. Comparative pathogenicity of nine US porcine reproductive and respiratory syndrome virus (PRRSV) isolates in a five-week-old cesarean-derived, colostrum-deprived pig model. J VET Diagn Invest 8:11–20. doi:10.1177/104063879600800103 9026065

[30] Zhao J, Xu Z, Xu T, Zhou Y, Li J, Deng H, Li F, Xu L, Sun X, Zhu L. 2022. Molecular characterization of the Nsp2 and ORF5S of PRRSV strains in Sichuan China during 2012–2020. Animals 12:3309. doi:10.3390/ani12233309 36496830 PMC9736255

[31] Zhao J, Zhu L, Huang J, Yang Z, Xu L, Gu S, Huang Y, Zhang R, Sun X, Zhou Y, Xu Z. 2021. Genetic characterization of a novel recombined porcine reproductive and respiratory syndrome virus 2 among Nadc30-like, Jxa1-like and TJ-like strains. Vet Med Sci 7:697–704. doi:10.1002/vms3.402 33277984 PMC8136965

[32] Wang FX, Song N, Chen LZ, Cheng SP, Wu H, Wen YJ. 2013. Non-structural protein 2 of the porcine reproductive and respiratory syndrome (PRRS) virus: a crucial protein in viral pathogenesis, immunity and diagnosis. Res Vet Sci 95:1–7. doi:10.1016/j.rvsc.2013.03.015 23591056

[33] Sun W, Wu W, Jiang N, Ge X, Zhang Y, Han J, Guo X, Zhou L, Yang H. 2022. Highly pathogenic PRRSV-infected alveolar macrophages impair the function of pulmonary microvascular endothelial cells. Viruses 14:452. doi:10.3390/v14030452 35336858 PMC8948932

[34] Chae C. 2021. Commercial PRRS modified-live virus vaccines. Vaccines 9:185. doi:10.3390/vaccines9020185 33671826 PMC7926738

[35] Risser J, Ackerman M, Evelsizer R, Wu S, Kwon B, Hammer JM. 2021. Porcine reproductive and respiratory syndrome virus genetic variability a management and diagnostic dilemma. Virol J 18:206. doi:10.1186/s12985-021-01675-0 34663367 PMC8522131

[36] Li P, Shen Y, Wang T, Li J, Li Y, Zhao Y, Liu S, Li B, Liu M, Meng F. 2022. Epidemiological survey of PRRS and genetic variation analysis of the ORF5 gene in Shandong province, 2020–2021. Front Vet Sci 9:987667. doi:10.3389/fvets.2022.987667 36187820 PMC9521713

[37] Zhou L, Yang Y, Xia Q, Guan Z, Zhang J, Li B, Qiu Y, Liu K, Shao D, Ma Z, Wang X, Wei J. 2022. Genetic characterization of porcine reproductive and respiratory syndrome virus from Eastern China during 2017-2022. Front Microbiol 13:971817. doi:10.3389/fmicb.2022.971817 36312912 PMC9606797

[38] Sha H, Zhang H, Chen Y, Huang L, Zhao M, Wang N. 2022. Research progress on the NSP9 protein of porcine reproductive and respiratory syndrome virus. Front Vet Sci 9:872205. doi:10.3389/fvets.2022.872205 35898550 PMC9309524

[39] Wang HM, Liu YG, Tang YD, Liu TX, Zheng LL, Wang TY, Liu SG, Wang G, Cai XH. 2018. A natural recombinant PRRSV between HP-PRRSV JXA1-like and NADC30-like strains. Transbound Emerg Dis 65:1078–1086. doi:10.1111/tbed.12852 29520988

[40] Zhou L, Kang R, Zhang Y, Yu J, Xie B, Chen C, Li X, Chen B, Liang L, Zhu J, Tian Y, Yang X, Wang H. 2019. Emergence of two novel recombinant porcine reproductive and respiratory syndrome viruses 2 (lineage 3) in southwestern China. Vet Microbiol 232:30–41. doi:10.1016/j.vetmic.2019.01.026 31030842

[41] Diao F, Jiang C, Sun Y, Gao Y, Bai J, Nauwynck H, Wang X, Yang Y, Jiang P, Liu X. 2023. Porcine reproductive and respiratory syndrome virus infection triggers autophagy via ER stress-induced calcium signaling to facilitate virus replication. PLoS Pathog 19:e1011295. doi:10.1371/journal.ppat.1011295 36972295 PMC10079224

[42] Vandenbussche F, Mathijs E, Tignon M, Vandersmissen T, Cay AB. 2021. WGS- versus ORF5-based typing of PRRSV: a Belgian case study. Viruses 13:2419. doi:10.3390/v13122419 34960688 PMC8707199

[43] Renukaradhya GJ, Meng XJ, Calvert JG, Roof M, Lager KM. 2015. Live porcine reproductive and respiratory syndrome virus vaccines: current status and future direction. Vaccine 33:4069–4080. doi:10.1016/j.vaccine.2015.06.092 26148878

[44] Li D-Y, Cui X-Y, Huang X-Y, Hu Y, Tian X-X, Wang T, Yang Y-B, Wang Q, Tian Z-J, Cai X-H, An T-Q. 2022. Characterization of two immunodominant antigenic peptides in NSP2 of PRRSV-2 and generation of a marker PRRSV strain based on the peptides. Front Vet Sci 9:902822. doi:10.3389/fvets.2022.902822 35706603 PMC9189411

[45] Zhao HZ, Wang FX, Han XY, Guo H, Liu CY, Hou LN, Wang YX, Zheng H, Wang L, Wen YJ. 2022. Recent advances in the study of NADC34-like porcine reproductive and respiratory syndrome virus in China. Front Microbiol 13:950402. doi:10.3389/fmicb.2022.950402 35935186 PMC9354828

[46] Ma J, Ma L, Yang M, Wu W, Feng W, Chen Z. 2021. The function of the PRRSV–host interactions and their effects on viral replication and propagation in antiviral strategies. Vaccines 9:364. doi:10.3390/vaccines9040364 33918746 PMC8070056

[47] Bian T, Sun Y, Hao M, Zhou L, Ge X, Guo X, Han J, Yang H. 2017. A recombinant type 2 porcine reproductive and respiratory syndrome virus between NADC30-like and a MLV-like: genetic characterization and pathogenicity for piglets. Infect Genet Evol 54:279–286. doi:10.1016/j.meegid.2017.07.016 28713014

[48] Liu J, Zhou X, Zhai J, Wei C, Dai A, Yang X, Luo M. 2017. Recombination in JXA1-R vaccine and NADC30-like strain of porcine reproductive and respiratory syndrome viruses. Vet Microbiol 204:110–120. doi:10.1016/j.vetmic.2017.04.017 28532789

[49] Zhao H, Han Q, Zhang L, Zhang Z, Wu Y, Shen H, Jiang P. 2017. Emergence of mosaic recombinant strains potentially associated with vaccine JXA1-R and predominant circulating strains of porcine reproductive and respiratory syndrome virus in different provinces of China. Virol J 14:67. doi:10.1186/s12985-017-0735-3 28376821 PMC5379541

[50] Su J, Zhou L, He B, Zhang X, Ge X, Han J, Guo X, Yang H. 2019. Nsp2 and GP5-M of porcine reproductive and respiratory syndrome virus contribute to targets for neutralizing antibodies. Virol Sin 34:631–640. doi:10.1007/s12250-019-00149-6 31347089 PMC6889258

[51] Music N, Gagnon CA. 2010. The role of porcine reproductive and respiratory syndrome (PRRS) virus structural and non-structural proteins in virus pathogenesis. Anim Health Res Rev 11:135–163. doi:10.1017/S1466252310000034 20388230

[52] Zheng Y, Zhang H, Luo Q, Sha H, Li G, Mu X, He Y, Kong W, Wu A, Zhang H, Yu X. 2023. Research progress on NSP11 of porcine reproductive and respiratory syndrome virus. Vet Sci 10:451. doi:10.3390/vetsci10070451 37505856 PMC10384725

[53] Chaudhari J, Liew C-S, Riethoven J-JM, Sillman S, Vu HLX. 2021. Porcine reproductive and respiratory syndrome virus infection upregulates negative immune regulators and T-cell exhaustion markers. J Virol 95:e0105221. doi:10.1128/JVI.01052-21 34379512 PMC8513478

[54] Li Y, Xu G, Du X, Xu L, Ma Z, Li Z, Feng Y, Jiao D, Guo W, Xiao S. 2021. Genomic characteristics and pathogenicity of a new recombinant strain of porcine reproductive and respiratory syndrome virus. Arch Virol 166:389–402. doi:10.1007/s00705-020-04917-8 33385245

