# Supplementary material for: Isolation and pathogenicity comparison of two novel natural recombinant porcine reproductive and respiratory syndrome viruses with different recombination patterns in Southwest China
Source: Microbiol Spectr. 2024 Mar 21;12(5):e04071-23. doi: 10.1128/spectrum.04071-23 (PMC11064529; doi:10.1128/spectrum.04071-23)
Supplement: Table S1 — The reference sequence information of PRRSV. [file spectrum.04071-23-s0001.doc]

**Supplementary Table S1**

The reference sequence information of PRRSV

| **No.** | **Virus Strain** | **Origin** | **Accession No.** | **No.** | **Virus Strain** | **Origin** | **Accession No.** |
| --- | --- | --- | --- | --- | --- | --- | --- |
| 1 | BJ-4 | China, 2016 | AF331831.1 | 22 | ISU18 | America, 2016 | KT257968.1 |
| 2 | BL2019 | China, 2019 | OQ735301.1 | 23 | JA142 | America, 2016 | AY424271.1 |
| 3 | CH-1a | China, 2016 | AY032626.1 | 24 | JL580 | China, 2015 | KR706343.1 |
| 4 | FJ0908 | China,2019 | MK202794.1 | 25 | JXA1 | China, 2016 | EF112445.1 |
| 5 | FJL15 | China, 2017 | KY412887.1 | 26 | Lelystad virus | Netherlands, 2019 | AY588319.1 |
| 6 | FJM4 | China, 2017 | KY412888.1 | 27 | LNCH-1604 | China, 2018 | MH651741.1 |
| 7 | FJSD | China, 2015 | KP998474.1 | 28 | LNDZD10-1806 | China, 2020 | MN648054.1 |
| 8 | FJY04 | China,2015 | KP860910.1 | 29 | LNWK130 | China, 2018 | MG913987.1 |
| 9 | FJZ03 | China, 2015 | KP860909.1 | 30 | MN184A | America, 2022 | DQ176019.1 |
| 10 | GM2 | China, 2012 | JN662424.1 | 31 | MN184B | America, 2022 | DQ176020.1 |
| 11 | HBFL-1604 | China, 2018 | MH651739.1 | 32 | MN184C | America, 2008 | EF488739.1 |
| 12 | HK14 | China, 2014 | KF287141.1 | 33 | NADC30 | America, 2019 | MH500776.1 |
| 13 | HLHDZD32-1901 | China, 2020 | MN648449.1 | 34 | NC/2014/ISU-3 | America, 2017 | MF326990.1 |
| 14 | HLJZD30-1902 | China,2020 | MN648055.1 | 35 | NL1207 | China, 2022 | MZ399800.1 |
| 15 | HNJYH-1606 | China, 2018 | MH651740.1 | 36 | NT1 | China, 2015 | KP179402.1 |
| 16 | HUN4 | China, 2016 | EF635006.1 | 37 | NT3 | China, 2015 | KP179404.1 |
| 17 | IA/2014/NADC34 | America, 2017 | MF326985.1 | 38 | QYYZ | China, 2012 | JQ308798.1 |
| 18 | IA/2015/ISU-9 | America, 2017 | MF326996.1 | 39 | SD176-1702 | China, 2022 | MT093751.1 |
| 19 | IA/2015/ISU-10 | America, 2015 | MF326997.1 | 40 | TJ | China, 2012 | EU860248.1 |
| 20 | IA/2015/ISU-14 | America, 2017 | MF327001.1 | 41 | VR2332 | America, 2018 | U87392 |
| 21 | ISU17 | America, 2016 | KT257967.1 |  |  |  |  |
